# *In Vitro* Selective Combinatory Effect of Ciprofloxacin with Nitroxoline, Sanguinarine, and Zinc Pyrithione against Diarrhea-Causing and Gut Beneficial Bacteria

**DOI:** 10.1128/spectrum.01063-22

**Published:** 2022-08-16

**Authors:** Hayford Osei-Owusu, Tomas Kudera, Marie Strakova, Johana Rondevaldova, Eva Skrivanova, Pavel Novy, Ladislav Kokoska

**Affiliations:** a Department of Crop Sciences and Agroforestry, Faculty of Tropical AgriSciences, Czech University of Life Sciences Prague, Prague-Suchdol, Czech Republic; b Department of Microbiology, Nutrition and Dietetics, Faculty of Agrobiology, Food and Natural Resources, Czech University of Life Sciences Prague, Prague-Suchdol, Czech Republic; c Department of Food Science, Faculty of Agrobiology, Food and Natural Resources, Czech University of Life Sciences Prague, Prague-Suchdol, Czech Republic; University of Exeter

**Keywords:** antagonism, antimicrobial agents, diarrhea, gut microbiota, selectivity, synergism

## Abstract

Antibiotic resistance in diarrhea-causing bacteria and its disruption of gut microbiota composition are health problems worldwide. The development of combinatory agents that increase the selective inhibitory effect (synergism) against diarrheagenic pathogens and, simultaneously, have a lowered impact (antagonism) or no negative action on the gut microbiota is therefore proposed as a new strategy efficient for chemotherapy against diarrheal conditions. In this study, the *in vitro* selective combinatory effect of ciprofloxacin with nitroxoline, sanguinarine, and zinc pyrithione, representing various classes of alkaloid-related compounds (nitroquinolines, benzylisoquinolines and metal-pyridine derivative complexes) against selected standard diarrhea-causing (Bacillus cereus, Enterococcus faecalis, Listeria monocytogenes, Shigella flexneri, and Vibrio parahaemolyticus) and gut-beneficial (Bifidobacterium adolescentis, Bifidobacterium animalis subsp. *lactis*, Bifidobacterium breve, Lactobacillus casei, and Lactobacillus rhamnosus) bacteria, was evaluated according to the sum of fractional inhibitory concentration indices (FICIs) obtained by the checkerboard method. The results showed that the individual combination of ciprofloxacin with nitroxoline, sanguinarine, and zinc pyrithione produced a synergistic effect against the pathogenic bacteria, with FICI values ranging from 0.071 to 0.5, whereas their antagonistic interaction toward the *Bifidobacterium* strains (with FICI values ranging from 4.012 to 8.023) was observed. Ciprofloxacin-zinc pyrithione produced significant synergistic action against S. flexneri, whereas a strong antagonistic interaction was observed toward B. breve for the ciprofloxacin-nitroxoline combination. These findings suggest that certain combinations of agents tested in this study can be used for the development of antidiarrheal therapeutic agents with reduced harmful action on the gastrointestinal microbiome. However, further studies focused on their pharmacological efficacy and safety are needed before they are considered for clinical trials.

**IMPORTANCE** Diarrheal infections, which are commonly treated by antibiotics, are still responsible for over 4 to 5 million cases of human deaths annually. Moreover, the rising incidence of antibiotic resistance and its negative effect on beneficial bacteria (e.g., *Bifidobacteria*) of the gut microbial community are another problem. Thus, the development of selective agents able to inhibit diarrheal bacteria and, simultaneously, that have no negative impact on the gut microbiota, is important. Our results showed that individual combinations of ciprofloxacin with nitroxoline, sanguinarine, and zinc pyrithione produced synergism against the pathogenic bacteria, whereas their antagonistic interaction toward the beneficial strains was observed. The antagonism can be considered a positive effect contributing to the safety of the therapeutic agents, whereas their synergism against diarrheal bacteria significantly potentiates total antimicrobial efficacy. The certain combinations tested in this study can be used for the development of antidiarrheal agents with reduced harmful action on the gastrointestinal microbiome.

## INTRODUCTION

Diarrheal infections and their complications are important causes of morbidity and mortality worldwide, and over 4 to 5 million cases of human deaths occur annually because of diarrheal infections (1). These infections are a problem especially in children younger than 2 years of age living in South Asia and sub-Saharan Africa (2). Besides a variety of viruses and parasites (3), Campylobacter jejuni, *Clostridium* spp., Escherichia coli, Salmonella spp., *Shigella* spp., Vibrio cholerae, and *Yersinia* spp. are examples of bacterial pathogens playing an important role in the etiology of human diarrheal infections (3, 4). Antimicrobial therapy using various antibiotics, such as ceftriaxone, chloramphenicol, ciprofloxacin, tetracycline, metronidazole, and vancomycin, is currently the common approach to treat acute infectious diarrhea (5). However, the indiscriminate and extensive use of these agents are associated with negative effects, such as the development of multidrug bacterial resistance (6) and dysbiosis of gut microbial community (7). It is well known that the ecosystem of the gastrointestinal tract is a repository of beneficial bacteria (e.g., *Bifidobacterium* and *Lactobacillus*), which are interacting with the epithelia of intestinal cells and strengthening the intestinal barrier against invasion and adherence of diarrheagenic microorganisms (8). Nowadays, various products containing beneficial bacteria are employed to treat digestive problems including diarrhea. For example, chewable tablets containing Lactobacillus acidophilus traded under the name Lactinex (Becton, Dickinson, and Company, Franklin Lakes, USA) are reported to be effective against antibiotic-associated diarrhea (9).

A combination therapy of two or more antibiotics is an effective strategy for the treatment of bacterial infections because it takes advantage of the additive effects of multiple antimicrobial mechanisms, improves clinical outcome, and lowers the risk of resistance development and mortality (10). Nowadays, combinations of β-lactam antibiotics with inhibitors of β-lactamases, for example, ampicillin with sulbactam (Unasyn, Pfizer, New York, USA), are used in pharmacological practice to treat intra-abdominal infections caused by β-lactamase-producing strains of Bacteroides fragilis, Enterobacter, E. coli, and Klebsiella (11). Although there are no combinatory therapies for the treatment of infectious diarrhea available at the market, previous clinical trials with various agents, such as antibiotics and/or microorganisms, showed promising results in this area. For example, in a placebo-controlled clinical trial, patients with Clostridium difficile*-*associated diarrhea responded well to a combination treatment of vancomycin or metronidazole and Saccharomyces boulardii (12). Also, it has been reported that trimethoprim-sulfamethoxazole with loperamide is very efficacious in the treatment of traveler’s diarrhea in a randomized, double-blind, placebo-controlled trial (13). The above-mentioned examples are based on the synergistic effect of two or more antibiotic agents that is increasing their efficacy. However, combinatorial approaches involving plant-derived compounds rather than antibiotics only might help alleviate the challenges of increasing antibiotic resistant because of their broader spectrum of mechanisms of antimicrobial actions, such as inhibiting bacterial cellular division and efflux pumps, intercalating bacterial DNA, and increasing cell membrane permeability (14, 15). Synergy of antimicrobials is defined as the interaction of two or more agents producing a lower dose or MIC that enhances each other’s activity, and their combined activity is greater than that expected by their individual potencies (16, 17). On the other hand, a decrease of the combinatory action, called antagonism (18), can be considered a positive effect on beneficial bacteria, and it occurs when one of the agents counteracts the action of the other, and thus, the combination of agents increases the MIC that is always less effective than the single agents at the same concentration (19). The development of agents selectively inhibiting the growth of diarrheal bacteria and, at the same time, having no negative impact on the gut microbiota can be an advanced strategy for safer and more efficient chemotherapy of diarrheal conditions. In view of this idea, experiments showing the selective growth-inhibitory effect of antidiarrheal agents have recently attracted the attention of numerous researchers. For example, studies of Novakova et al. (20, 21) have demonstrated the *in vitro* selectivity effect of 8-hydroxyquinoline, a quinolone alkaloid of plant origin, toward some diarrheagenic bacteria (e.g., C. difficile) with lower effect on some of the beneficial ones (e.g., bifidobacteria). Another experiment analyzed in media containing chicken ileal digesta has revealed that 8-hydroxyquinoline exhibits an *in vitro* selective growth-inhibitory effect against C. perfringens over bifidobacterial strains tested (22). Additionally, the work by Sklenickova et al. (23) has shown a selective effect of biochanin A against enteropathogenic (e.g., *Clostridium* spp.) and beneficial (e.g., *Bifidobacterium* spp.) bacteria. However, studies focused on the identification of agents producing both combinatory and selective antimicrobial actions are completely missing.

According to the recently published studies, conventional antibiotics and plant-derived compounds seem to be promising agents for research on their synergistic growth-inhibitory activity against diarrhea-causing microorganisms combined with the selectivity toward pathogenic and beneficial gut microbiota. For example, ciprofloxacin, a broad-spectrum fluoroquinolone antibiotic commonly used in the treatment of diarrhea (24, 25), has shown a significant *in vitro* growth-inhibitory effect against clostridia (C. difficile and C. perfringens) when combined with metronidazole (26). Furthermore, ciprofloxacin has been reported to exhibit selective antibacterial activity against diarrhea-causing pathogens (e.g., S. flexneri) with a lower effect on beneficial strains (e.g., B. breve) (27). Among various classes of antimicrobial, active plant-derived compounds, alkaloids appear to be prospective candidates producing synergistic and selective antibacterial effects. For example, sanguinarine, a benzylisoquinoline alkaloid isolated from *Sanguinaria canadensis* (28), has been reported to show a synergistic effect in combination with streptomycin against clinical isolates of E. coli (29). In our recent study, nitroxoline (a drug used to treat infectious and inflammatory diseases of the urogenital tract [30, 31] that was derived from the molecule of 8-hydroxyquinoline detected previously in Centaurea diffusa [32]) and zinc pyrithione (a metal complex of pyrithione, an organosulfur compound found in Polyalthia nemoralis [33], that is used in cosmetic and shampoo products for external application against skin and hair infections, such as dandruff caused by *Malassezia* spp. [34, 35]) have demonstrated a significant selective *in vitro* growth-inhibitory effect against diarrheic bacteria with minimal action toward the beneficial ones (27). However, the selective combinatory effect of above-mentioned compounds toward representatives of gut diarrheagenic and beneficial bacteria has never been researched. Therefore, in this study, we decided to evaluate combinations of ciprofloxacin with nitroxoline, sanguinarine, and zinc pyrithione for their synergistic effect against diarrheal pathogens (B. cereus, E. faecalis, L. monocytogenes, S. flexneri, and V. parahaemolyticus) and antagonism toward bifidobacteria (B. adolescentis, *B. animalis* subsp. *lactis*, and B. breve) and lactobacilli (*L. casei* and L. rhamnosus).

## RESULTS

In this study, combinations of ciprofloxacin with nitroxoline, sanguinarine, and zinc pyrithione produced synergistic activity against diarrhea-causing bacteria, and at the same time, their antagonistic action decreased the potential harmful effects of these agents on gut beneficial bacteria. Among all antimicrobial agents tested, the combination of ciprofloxacin with zinc pyrithione produced the most significant synergistic action (FICI, 0.071) against the diarrhea-causing pathogens, whereas the strongest antagonism on gut beneficial bacteria was demonstrated by nitroxoline when combined with ciprofloxacin (FICI, 8.023). Based on FICIs, S. flexneri was the most susceptible bacterium to the combination of ciprofloxacin with zinc pyrithione, while the growth of B. breve was least affected by active concentrations of the nitroxoline and ciprofloxacin combination.

For the combination of ciprofloxacin with nitroxoline, several synergistic interactions were observed for all five standard diarrheagenic bacteria tested, with FICI values ranging from 0.094 to 0.477. The strongest synergy (FICI, 0.094) was obtained against L. monocytogenes at a nitroxoline concentration of 0.25 μg/mL, when a 13-fold ciprofloxacin MIC decrease was achieved (from 1.333 to 0.104 μg/mL). Furthermore, the highest ciprofloxacin MIC reduction was observed for V. parahaemolyticus, where the nitroxoline concentration of 1 μg/mL caused a 16-fold reduction of ciprofloxacin MIC from 0.125 to 0.008 μg/mL, with a FICI of 0.313. In the case of the gut beneficial bacteria, the mixtures of these antimicrobial agents revealed a clear antagonistic effect on three of five species tested, with FICI values ranging from 4.012 to 8.023. The significant antagonistic action (FICI, 8.023) was observed toward B. breve at a ciprofloxacin concentration of 0.125 μg/mL, when 8-fold MIC increase of nitroxoline was achieved (from 4 to 32 μg/mL). Also, *B. animalis* subsp. *lactis* showed high resistance to this combination where the ciprofloxacin MIC of 10.667 μg/mL caused an 8-fold rise in the inhibitory concentration of nitroxoline (from 4 to 32 μg/mL) to produce a FICI value of 8.012. No interaction relationship was observed when these agents were combined against *L*. *casei* and L. rhamnosus. The results summarized in Table 1 show the complete data on growth-inhibitory activities of the tested ciprofloxacin with nitroxoline against diarrheagenic and beneficial bacteria, including calculated FICI values.

**TABLE 1:** *In vitro* susceptibilities of diarrhea-causing and gut beneficial bacteria to ciprofloxacin and nitroxoline alone and in combination

Bacterium	MIC^*a*^ of:		CIP results by the NTX MIC of:^*b*^																			
	CIP	NTX	32		16		8		4		2		1		0.5		0.25		0.125		0.063	
			MIC	FICI	MIC	FICI	MIC	FICI	MIC	FICI	MIC	FICI	MIC	FICI	MIC	FICI	MIC	FICI	MIC	FICI	MIC	FICI
Beneficial bacteria																						
B. adolescentis	4	8	0.063	4.016	0.063	2.016	0.063	1.016	4	1.5	4	1.25	4	1.125	4	1.063	4	1.031				
*B. animalis* subsp. *lactis*	10.667	4	0.125	8.012	0.125	4.012	0.125	2.012	9.333	1.875	10.667	1.5	10.67	1.25	10.667	1.125	10.667	1.063				
B. breve	5.333	4	0.125	8.023	0.125	4.023	0.125	2.023	0.75	1.141	5.333	1.5	5.333	1.25	5.333	1.125	5.333	1.063				
*L. casei*	1.333	16	0.063	2.047	0.063	1.047	1.333	1.5	1.333	1.25	2	1.625	2	1.563	2	1.532	2	1.516				
L. rhamnosus	2	16	0.063	2.031	0.063	1.031	2	1.5	3.333	1.917	3.333	1.792	3.333	1.729	3.333	1.698	3.333	1.682				
Diarrheal bacteria																						
E. faecalis	0.5	16	0.016	2.031	0.016	1.031	0.016	0.531	0.135	0.521	0.125	0.375	0.125	0.313	0.125	0.281	0.125	0.266				
L. monocytogenes	1.333	16	0.016	2.012	0.016	1.012	0.016	0.512	0.5	0.625	0.416	0.437	0.833	0.687	0.5	0.406	0.104	0.094				
B. cereus	0.5	6.667					0.016	1.231	0.057	0.715	0.089	0.477	0.094	0.338	0.094	0.263	0.125	0.287	0.25	0.519	0.25	0.509
S. flexneri	0.016	2					0.002	4.125	0.002	2.125	0.002	1.125	0.008	1	0.008	0.75	0.008	0.625	0.008	0.563	0.004	0.281
V. parahaemolyticus	0.125	4					0.008	2.063	0.008	1.063	0.008	0.563	0.008	0.313	0.063	0.625	0.063	0.563	0.063	0.531	0.063	0.516

a MIC, MIC of ciprofloxacin (CIP) and nitroxoline (NTX) expressed as an average from three independent experiments, with each performed in triplicate. Units for all MICs are μg/mL.

b FICI, fractional inhibitory concentration index; FICI values of ≤0.5 indicate synergistic effects, FICI values of >0.5–4 indicate no interaction effect, and FICI values of >4 indicate an antagonistic effect.

The combination of ciprofloxacin-sanguinarine exerted a growth-inhibitory interaction against the five diarrhea-causing pathogens tested, with the synergistic effect ranging between FICI values of 0.134 and 0.5. It also exhibited antagonism toward two out of five beneficial bacteria tested in this study, with FICI ranging from 4.012 to 8.016. Moreover, no interaction relationship was observed when these agents were combined against *L*. *casei* and L. rhamnosus. The most significant synergistic effect with a FICI value of 0.134 was documented against E. faecalis at a sanguinarine concentration of 0.25 μg/mL, when an 8-fold ciprofloxacin MIC decrease was achieved from 0.5 to 0.063 μg/mL. B. cereus was another diarrhea-causing bacterium significantly affected by the combination of these agents. In this case, ciprofloxacin at a concentration of 0.25 μg/mL caused a decline in the sanguinarine MIC by 32-fold (from 64 to 2 μg/mL) resulting in a FICI value of 0.198. Additionally, the highest ciprofloxacin MIC reduction was achieved for L. monocytogenes, where a sanguinarine concentration of 2 μg/mL produced a 104-fold reduction of ciprofloxacin MIC from 1.667 to 0.016 μg/mL with a FICI of 0.259. Based on the FICI evaluation, the above-mentioned agents demonstrated the strongest antagonistic effect toward B. adolescentis, at a ciprofloxacin concentration of 4 μg/mL, when an 8-fold MIC increase of sanguinarine was achieved (from 4 to 32 μg/mL) with a FICI value of 8.016. This result was followed by B. breve which showed resistance to the combination with a FICI of 4.023, where the ciprofloxacin MIC of 5.333 μg/mL caused a 4-fold rise in the active concentration of sanguinarine (from 8 to 32 μg/mL). The individual MICs of ciprofloxacin and sanguinarine against diarrhea-causing and beneficial bacteria as well as the MICs of their combinations with corresponding FICI values are summarized in Table 2.

**TABLE 2:** *In vitro* susceptibilities of diarrhea-causing and gut beneficial bacteria to ciprofloxacin and sanguinarine alone and in combination

Bacterium	MIC^*a*^ of:		CIP results by the MIC SNG of:^*b*^																							
	CIP	SNG	256		128		64		32		16		8		4		2		1		0.5		0.25		0.125	
			MIC	FICI	MIC	FICI	MIC	FICI	MIC	FICI	MIC	FICI	MIC	FICI	MIC	FICI	MIC	FICI	MIC	FICI	MIC	FICI	MIC	FICI	MIC	FICI
Beneficial bacteria																										
B. adolescentis	4	4							0.063	8.016	0.063	4.016	0.063	2.016	0.063	1.016	4	1.5	4	1.25	4	1.125	4	1.063		
*B. animalis* subsp. *lactis*	8	13.333							0.125	2.416	0.125	1.216	8.042	1.605	16	2.300	16	2.15	16	2.075	16	2.038	10.67	1.352		
B. breve	5.333	8							0.125	4.023	0.125	2.023	1.417	1.266	1.417	0.766	5.333	1.25	5.333	1.125	5.333	1.063	5.333	1.031		
*L. casei*	2	16							0.063	2.031	0.063	1.031	2.333	1.667	4	2.25	4	2.125	4	2.063	4	2.031	4	2.016		
L. rhamnosus	3.333	32							0.063	1.019	2	1.100	2.667	1.050	2.667	0.925	2.667	0.863	2.667	0.831	2.667	0.816	3.333	1.008		
Diarrheal bacteria																										
E. faecalis	0.5	26.667							0.016	1.231	0.016	0.631	0.016	0.331	0.016	0.181	0.094	0.263	0.125	0.287	0.125	0.269	0.063	0.134		
S. flexneri	0.016	16							0.002	2.125	0.002	1.125	0.002	0.625	0.008	0.750	0.008	0.625	0.008	0.563	0.008	0.531	0.004	0.266		
V. parahaemolyticus	0.125	32							0.004	1.032	0.004	0.532	0.007	0.306	0.042	0.461	0.063	0.567	0.083	0.695	0.104	0.848	0.125	1.008		
L. monocytogenes	1.667	8									0.016	2.009	0.016	1.009	0.016	0.509	0.016	0.259	0.339	0.328	0.833	0.562	0.833	0.531	1	0.616
B. cereus	0.25	64	0.016	4.063	0.016	2.063	0.016	1.063	0.063	0.75	0.063	0.5	0.063	0.375	0.063	0.313	0.042	0.198								

a MIC, MIC of ciprofloxacin (CIP) and sanguinarine (SNG) expressed as an average from three independent experiments, with each performed in triplicate. Units for all MICs are μg/mL.

b FICI, fractional inhibitory concentration index; FICI values of ≤0.5 indicate synergistic effects, FICI values of >0.5–4 indicate no interaction effect, and FICI values >4 indicate an antagonistic effect.

In reference to the data summarized in Table 3, the combination of ciprofloxacin with zinc pyrithione produced a synergistic antimicrobial effect (FICI ranging from 0.071 to 0.5) against all the pathogenic bacteria. The strongest synergistic activity with a FICI value of 0.071 was achieved against S. flexneri at a zinc pyrithione concentration of 0.125 μg/mL, when a 17-fold ciprofloxacin MIC decrease was attained (from 0.083 to 0.005 μg/mL). In addition, the highest ciprofloxacin MIC reduction was observed for L. monocytogenes, where a zinc pyrithione concentration of 2 μg/mL caused a 63-fold decrease of ciprofloxacin MIC from 1 to 0.016 μg/mL with a FICI of 0.316. Considering the gut beneficial microbes assessed in this work, the combinations of ciprofloxacin and zinc pyrithione showed antagonistic action toward B. breve at a ciprofloxacin concentration of 0.125 μg/mL, when a 5-fold MIC increase of zinc pyrithione was achieved from 13.333 to 64 μg/mL with FICI a value of 4.832. A mixture of both agents exhibited no interaction on *L*. *casei* and L. rhamnosus.

**TABLE 3:** *In vitro* susceptibilities of diarrhea-causing and gut beneficial bacteria to ciprofloxacin and zinc pyrithione alone and in combination

Bacterium	MIC^*a*^ of:		CIP results by the ZPT MIC of:^*b*^																							
	CIP	ZPT	64		32		16		8		4		2		1		0.5		0.25		0.125		0.063		0.031	
			MIC	FICI	MIC	FICI	MIC	FICI	MIC	FICI	MIC	FICI	MIC	FICI	MIC	FICI	MIC	FICI	MIC	FICI	MIC	FICI	MIC	FICI	MIC	FICI
Beneficial bacteria																										
B. adolescentis	4	64			0.063	0.516	4	1.25	4	1.125	4	1.063	4	1.031	4	1.016	4	1.008	4	1.004						
*B. animalis* subsp. *lactis*	9.333	22.667			0.125	1.425	5.375	1.282	5.375	0.929	5.375	0.752	6.667	0.803	8	0.901	8	0.879	8	0.868						
B. breve	4	13.333	0.125	4.832	0.125	2.432	2.333	1.784	3.333	1.433	3.333	1.133	4	1.15	4	1.075	4	1.038								
*L. casei*	1	32	0.063	2.063	0.063	1.063	0.354	0.854	0.5	0.75	0.833	0.958	0.833	0.896	0.833	0.864	0.833	0.849								
L. rhamnosus	1.666	32	0.063	2.038	0.063	1.038	0.125	0.575	1	0.85	2	1.325	2	1.263	2	1.232	2	1.216								
Diarrheal bacteria																										
L. monocytogenes	1	6.667									0.016	0.616	0.016	0.316	0.25	0.4	0.25	0.325	0.333	0.370	0.417	0.436	0.375	0.384	0.125	0.130
B. cereus	0.417	4							0.016	2.037	0.016	1.037	0.037	0.588	0.037	0.338	0.073	0.300	0.094	0.287	0.167	0.431	0.104	0.266		
E. faecalis	0.5	8							0.016	1.031	0.016	0.531	0.125	0.5	0.25	0.625	0.125	0.313	0.125	0.281	0.125	0.266	0.125	0.258		
S. flexneri	0.083	16					0.002	1.024	0.002	0.524	0.006	0.321	0.007	0.203	0.007	0.141	0.007	0.110	0.026	0.329	0.005	0.071				
V. parahaemolyticus	0.063	8					0.008	2.124	0.008	1.124	0.008	0.624	0.016	0.498	0.063	1.117	0.063	1.055	0.104	1.685	0.063	1.008				

a MIC, MIC of ciprofloxacin (CIP) and zinc pyrithione (ZPT) expressed as an average from three independent experiments each performed in triplicate. Units for all MICs are μg/mL.

b FICI, fractional inhibitory concentration index; FICI values (≤0.5) indicate synergistic effects; FICI values (>0.5 – 4) indicate no interaction effect; FICI values (>4) indicate antagonistic effect.

**FIG 1:**
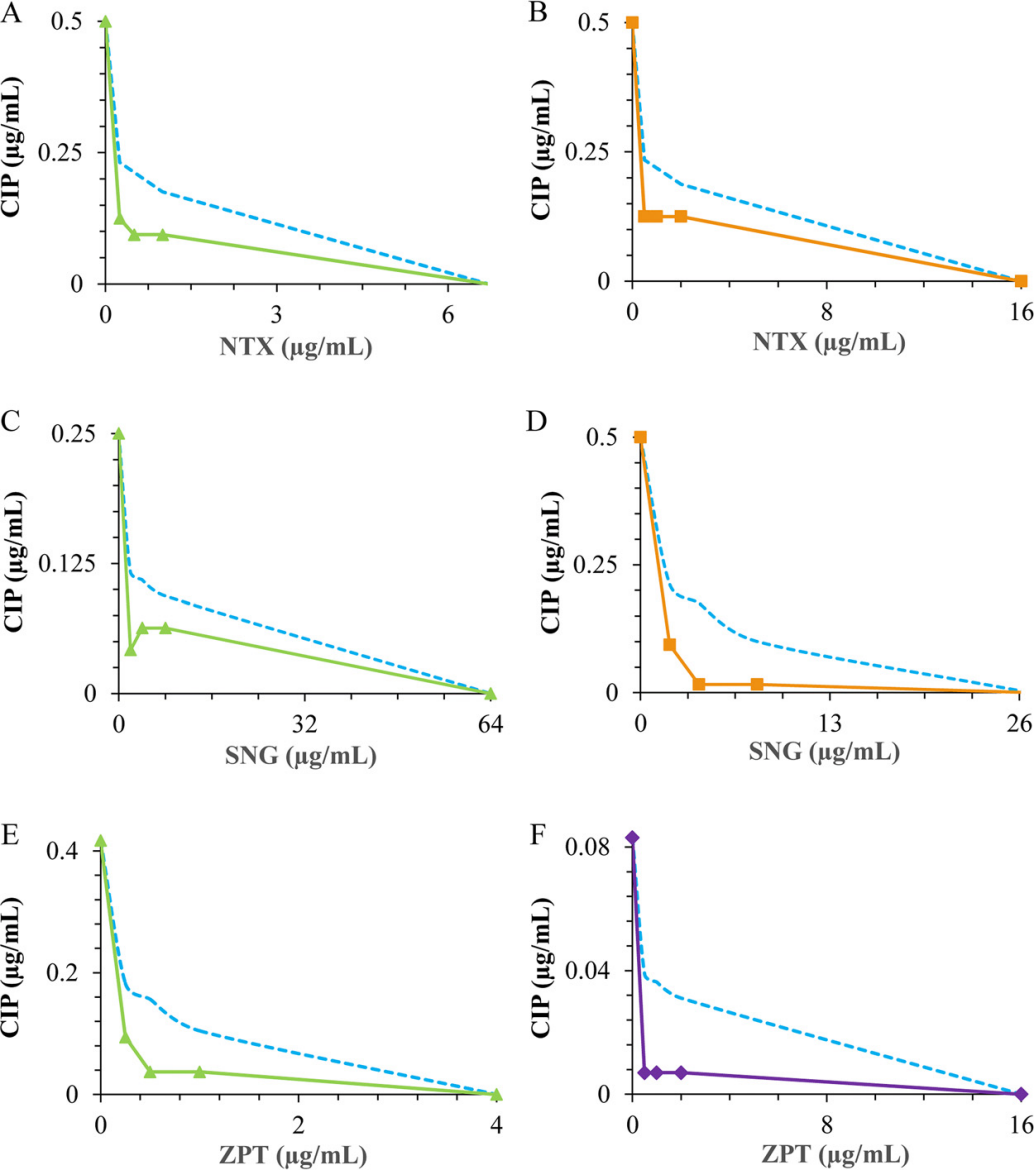
Isobolograms of the synergistic interactions for ciprofloxacin (CIP) with nitroxoline (NTX) against Bacillus cereus (A) and Enterococcus faecalis (B), sanguinarine (SNG) against B. cereus (C) and E. faecalis (D), and zinc pyrithione (ZPT) against B. cereus (E) and Shigella flexneri (F). ▴, B. cereus; ■, E. faecalis; ♦, S. flexneri; -, border for synergy.

**FIG 2:**
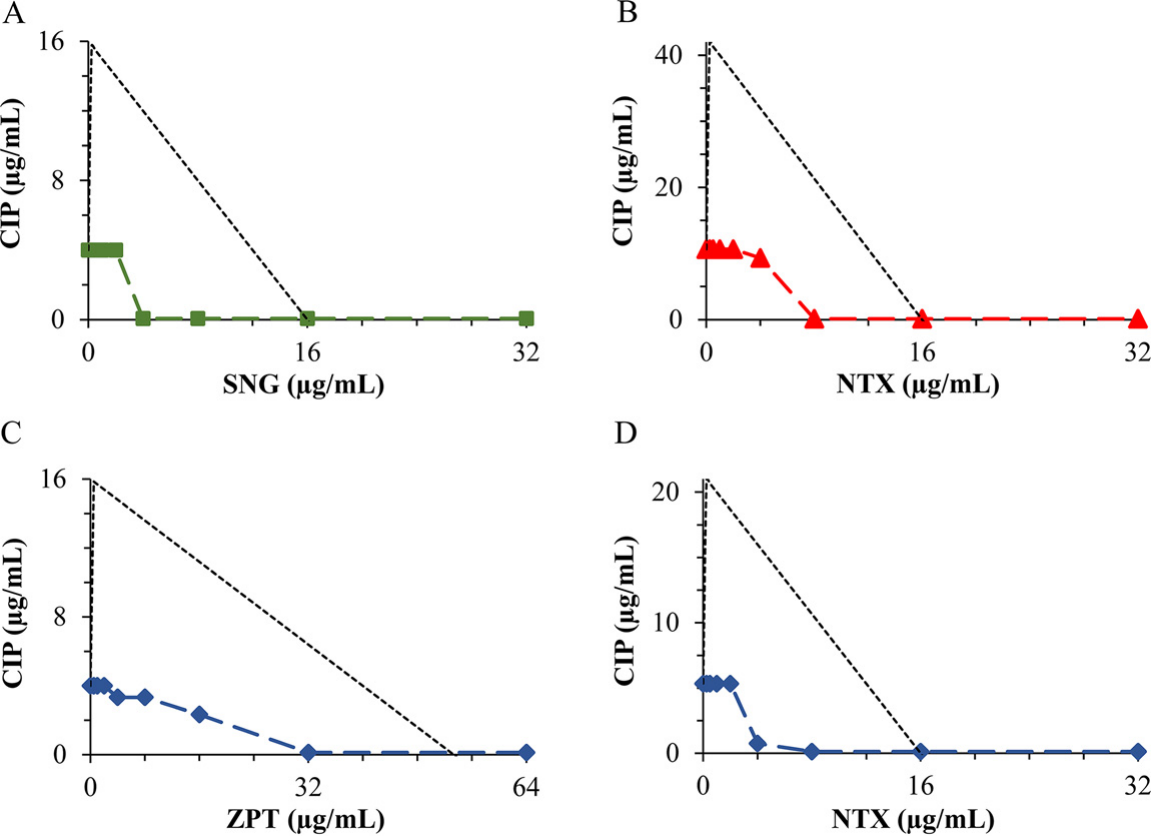
Isobolograms of the antagonistic interactions for ciprofloxacin (CIP) with sanguinarine (SNG) against Bifidobacterium adolescentis (A), nitroxoline (NTX) against *B. animalis* subsp. *lactis* (B) and B. breve (D), and zinc pyrithione (ZPT) against B. breve (C). ■, B. adolescentis; ▴, *B. animalis* subsp. *lactis*; ♦, B. breve; -, border for antagonism.

The combination profiles of the most sensitive and resistant bacteria are presented graphically in the form of isobologram curves (Fig. 1 and 2), which represent the results of the checkerboard assay and the FICI values, whereas the axes of each isobologram are the dose-axes of the individual agents. The resulting isobolograms confirmed the synergistic effect of ciprofloxacin in combination with nitroxoline, sanguinarine, and zinc pyrithione against B. cereus, E. faecalis, and S. flexneri, where synergy was observed for three ratios in the isobolograms of each strain. Also, the antagonistic effect of the ciprofloxacin-zinc pyrithione combination was observed toward B. breve for one ratio, whereas ciprofloxacin in combination with nitroxoline and sanguinarine showed two ratios toward B. adolescentis, B. breve, and *B. animalis* subsp*. lactis*. In this regard, an upward concave curve (light-green, orange, and purple solid lines) represents the confirmation of antimicrobial synergy observed against the diarrhea-causing bacteria, whereas a downward concave curve (deep-green, red, and blue dashed lines) distinctively suggests antagonism achieved toward bifidobacteria.

## DISCUSSION

In accordance with the standardized antimicrobial susceptibility testing breakpoint data (36), the MIC range of ciprofloxacin (0.016 to 1 μg/mL) observed in this study for pathogenic bacteria indicates their susceptibility to this antibiotic. Its previously reported MIC values against B. cereus, E. faecalis, L. monocytogenes, S. flexneri, and V. parahaemolyticus were in ranges of 0.03 to 1, 0.25 to 1, 1 to 4, 0.008 to 0.03, and 0.20 to 0.39 μg/mL (37–41), respectively; these findings correspond well with the results of our experiments. In the case of beneficial bacteria, Masco et al. (42) has reported ciprofloxacin MIC values ranging from 1 to 16 μg/mL toward the standard strains of bifidobacteria, which is similar to data recorded in this study. Moreover, consistent with our findings, Rozman et al. (43) reported MIC values of 1 and 2 μg/mL toward *L*. *casei* and L. rhamnosus, respectively. In another study conducted by Chang et al. (44), ciprofloxacin produced MIC values ranging from 4 to 64 μg/mL against *L*. *casei* isolated from fecal samples of children. Compared with our report, this discrepancy may be caused by the different susceptibility of the standard bacterial strain used in this study. According to the studies published recently, alkaloid-related agents (nitroxoline, sanguinarine, and zinc pyrithione) have demonstrated a growth-inhibitory effect against the diarrhea-causing bacteria tested, at MIC values ranging from 1 to 128 μg/mL. In addition, the study further reported MIC values of the above-mentioned agents toward the standard gut beneficial bacteria ranging from 16 to 512 μg/mL (27). The MIC values recorded in our study belong to this range. It is therefore suggested that our result describes the bacteriostatic effect (bacterial growth inhibition) of the antibacterial agents tested rather than its bactericidal effect (kill bacterial growth) (45), which may provide valuable information in the area of pharmacological research. However, further research on the nature of bacterial growth inhibitory effects of these antimicrobial agents identified in this study is required. The synergistic effect of ciprofloxacin with other antibiotics (e.g., gentamicin and trimethoprim) has been reported previously against clinical isolates of bacterial pathogens causing diarrhea (46, 47). Furthermore, another study has shown that the combination of this antibiotic with isoquinoline alkaloid berberine produced synergistic action against strain species of diarrheagenic bacteria (48). Nevertheless, experimental data on the combined effect of ciprofloxacin with nitroxoline, sanguinarine, and zinc pyrithione are completely missing. Recently, a paper on the selective antibacterial action of the above-mentioned agents against diarrheic and beneficial gut bacteria appeared in the literature (27). In our investigation, ciprofloxacin in combination with nitroxoline, sanguinarine, and zinc pyrithione at various inhibitory concentrations produced significant synergism against diarrheal pathogens as well as antagonism on bifidobacteria. To the best of our knowledge, this is the first report demonstrating the selective combinatory effect of antimicrobial agents against diarrhea-causing and gut-beneficial bacteria.

From the results presented in this study, it is not possible to conclude what action mechanisms are responsible for synergistic and antagonistic interactions between ciprofloxacin and the tested agents. However, based on literature data, it can be hypothesized that different mechanisms are responsible for the ability of nitroxoline, sanguinarine, and zinc pyrithione to reduce the MIC of ciprofloxacin against the diarrheagenic bacteria as well as for the capacity of ciprofloxacin to increase the MICs of the earlier-mentioned agents toward the beneficial bacteria. It has been documented that ciprofloxacin targets topoisomerase enzymes essential for bacterial DNA synthesis, namely, DNA gyrase and topoisomerase IV (49). Metal ion cofactors (e.g., magnesium ion) are known to substantially affect the biological properties of topoisomerases (50). It has previously been described that the activity of the quinolones is reduced in the presence of divalent cations, such as Mg^2+^ (51). Since nitroxoline and zinc pyrithione have been observed to chelate metal ions (52, 53), it is possible to assume that Mg^2+^ bonded to these compounds is not available as a cofactor necessary for proper functions of topoisomerases, and, at the same time, it does not affect the activity of ciprofloxacin. The ability of both agents to form complexes with Mg^2+^ may therefore significantly contribute to their synergistic antibacterial action with ciprofloxacin observed in this study. Regarding the antagonistic activity observed in this study, we assume that the ability of bifidobacteria to sequester and accumulate metal ions from their environment (54) can contribute to its increased resistance to nitroxoline and zinc pyrithione in the presence of ciprofloxacin. It can also be hypothesized that ciprofloxacin, which increases bacterial membrane permeability by the release of bound Ca^2+^ into the cytosol (55), can enhance the penetration of sanguinarine, which blocks cytokinesis in bacteria by inhibiting Z-ring formation (56), into the bacterial cells. On the other hand, sanguinarine may also enhance the intracellular penetration of ciprofloxacin because it has been observed that the permeability of the bacterial cell membrane increases in the presence of this alkaloid (57, 58). This information may be suggested as a probable mechanism underlying the synergistic antibacterial interaction between these two antimicrobial agents against the diarrheal-causing bacteria examined in our studies. Although the presence of sanguinarine in the feed administered to experimental animals has previously been observed to increase the levels of bifidobacteria in the contents of their intestinal lumina (59), the mechanism responsible for the increased resistance of this bacteria to sanguinarine in the presence of ciprofloxacin is unclear. Interestingly, ciprofloxacin MICs were greatly reduced at high concentrations of the second compound, whereas those concentrations were in many cases above the MIC of the second agent. The similar phenomenon was observed in study of Lalouckova et al. (60), who researched the antagonistic effect of oxacillin in combination with palm seed crude oils and lauric acid against Staphylococcus aureus.

Since both ciprofloxacin and nitroxoline are drugs commonly used in clinical practice for the treatment of bacterial infections (61, 62) the use of their combination could be a potential treatment strategy against several fluoroquinolone-resistant infections of the gastrointestinal tract, such as ciprofloxacin-resistant shigellosis which has been a recurrent challenge in many parts of developing world (63). Since sanguinarine has been observed to be a slightly toxic substance when administered orally to rats (median lethal dose [LD_50_], 1,658 mg/kg of body weight) (64), its pharmacological use seems to be limited. When administered orally, zinc pyrithione is classified as a moderately toxic agent with LD_50_ values ranging from 92 to 266 mg/kg in rats and from 160 to 1,000 mg/kg in mice (65). Although zinc pyrithione is an antifungal ingredient well-known in cosmetic and shampoo products, its use in the form of an orally administered agent therefore seems to be even less realistic than the use of sanguinarine. However, it is possible to suppose that the significantly lowered active concentrations of sanguinarine and zinc pyrithione resulting from their antibacterial synergy with ciprofloxacin can produce a lower toxicological response in target organisms. Also, further toxicological studies are needed to examine the therapeutic safety of ciprofloxacin combinations with above-mentioned agents before their possible pharmacological usage.

In summary, the present study has demonstrated that combinations of ciprofloxacin with nitroxoline, sanguinarine, and zinc pyrithione produce an antibacterial synergistic effect against diarrhea-causing bacteria, namely, B. cereus, E. faecalis, L. monocytogenes, S. flexneri, and V. parahaemolyticus, and, simultaneously, has showed antagonistic action toward gut beneficial strains of bifidobacteria, such as B. adolescentis, *B. animalis* subsp. *Lactis*, and B. breve. For the first time, this study demonstrates a selective combinatory effect of antimicrobial agents against diarrhea-causing and gut beneficial bacteria. The observed antagonism of tested agents toward gut microbiota can be considered a positive effect contributing to the safety of the therapeutic agents, whereas their synergism against diarrheal bacteria significantly potentiates total antimicrobial efficacy. The results suggest that certain combinations of agents tested in this study can be used for the development of antidiarrheal therapeutic agents with reduced harmful action on the gastrointestinal microbiome. However, further studies that focus on their *in vivo* antibacterial activity and safety are needed before any consideration for clinical trials. Furthermore, investigations concerning the exact mechanism of their selective combinatory actions should be conducted.

## MATERIALS AND METHODS

### Chemicals.

Phytochemicals (sanguinarine chloride), synthetic analogs of natural products (nitroxoline and zinc pyrithione), and antibiotics (ciprofloxacin) used in this study were purchased from Sigma-Aldrich (Prague, Czech Republic). Dimethyl sulfoxide (DMSO) (Sigma-Aldrich) was used to prepare the stock solutions of all test compounds except ciprofloxacin, which was prepared using distilled water.

### Bacterial strains and growth media.

The intestinal bacterial type strains were obtained from the American Type Culture Collection (ATCC; Rockville, USA), Czech Collection of Microorganisms (CCM; Brno, Czech Republic), and German Collection of Microorganisms and Cell Cultures (DSMZ; Braunschweig, Germany). The following five strains, which included Gram-positive and Gram-negative bacteria that are representative of diarrheagenic pathogens associated with globally distributed foodborne, waterborne, and nosocomial infections, were used in this study: B. cereus ATCC 14579, E. faecalis ATCC 29212, L. monocytogenes ATCC 7644, S. flexneri ATCC 12022, and V. parahaemolyticus ATCC 17802. In addition, the following five bacterial strains, which are among the three predominant bacterial phyla in the human gut and exhibit beneficial functions, were used in this study: B. adolescentis DSMZ 20087, *B. animalis* subsp. *lactis* DSMZ 10140, B. breve ATCC 15700, *L. casei* DSMZ 20011, and L. rhamnosus CCM 7091. As the maintenance and growth medium, Mueller-Hinton broth (Oxoid, Basingstoke, UK) was used for bacteria that grow aerobically (for V. parahaemolyticus supplemented by 3% NaCl). The anaerobic bacteria (bifidobacteria), including facultative species (lactobacilli), were cultured in Wilkins-Chalgren broth (Oxoid) supplemented with 5 g/L soya peptone and 0.5 g/L cysteine.

### MIC determination and evaluation of the combined antimicrobial effect.

The *in vitro* growth-inhibitory activities of the compounds tested against aerobic and anaerobic bacterial strains were evaluated by the broth microdilution method using 96-well microtiter plates, following the protocols of Clinical and Laboratory Standards Institute (CLSI) guidelines (66) and Hecht et al. (67), respectively. The combinatory effect of ciprofloxacin with nitroxoline, sanguinarine chloride, and zinc pyrithione against pathogenic and beneficial bacteria was evaluated by the checkerboard method based on fractional inhibitory concentration indices (FICIs) (68). These tests were modified according to the recommendations proposed for an effective assessment of the anti-infective potential of natural products by Cos et al. (69), whereby MIC values used for FICI calculation were determined using spectrophotometric measurement of bacterial growth (optical density). Prior to testing, the strains were subcultured in appropriate media at 37°C for 24 h. Obligate anaerobes and lactobacilli were cultured for 48 h using the Whitley A35 anaerobic workstation (Don Whitley Scientific, Bingley, UK). The anaerobic conditions were created by the supply of standard anaerobic gas mixture of 10% H_2_, 10% CO_2_, and 80% N_2_ (Linde Gas, Prague, Czech Republic).

In combinations, eight 2-fold serial dilutions of antibiotic ciprofloxacin from horizontal rows of the microtiter plate were subsequently cross-diluted vertically by eight 2-fold serial dilutions of the test compound (nitroxoline, sanguinarine, or zinc pyrithione). Assay microplate preparation and serial dilution for the growth of aerobic and anaerobic bacteria were performed in appropriate growth media using the Freedom EVO 100 automated pipetting platform (Tecan, Mannedorf, Switzerland) and manually with a multichannel pipette (Eppendorf, Hamburg, Germany) respectively. The initial concentration for ciprofloxacin was optimized depending on the susceptibility of the bacterial strains tested, whereas that of the rest of the agents was 512 μg/mL. The subcultured bacterial density of 1.5 × 10^8^ CFU/mL adjusted by 0.5 McFarland standard using a Densi-La-Meter II instrument (Lachema, Brno, Czech Republic) was used to inoculate the 96-well plates (5 μL/well). Bacterial cultures in microplates were incubated by employing the same protocols as used for their cultivation prior to the test. The optical density of the cultures was measured at 405 nm using a Cytation 3 imaging reader (BioTek, Winooski, USA) after the growth (69). The lowest concentration (μg/mL) of test compounds at which the bacterial growth was inhibited by ≥80% was defined as the MIC (70). The MICs presented in this work were the average of MICs obtained from three independent experiments, with each performed in triplicate (71). The highest concentration of DMSO present in the microtiter plates (1%) did not inhibit the bacterial growth of any strain tested.

The combined effects of the antibiotics (A) with the tested compounds (B) were then determined based on sum of FIC values, which was calculated according to the following equation: ∑FIC = FIC_A_ + FIC_B_, where FIC_A_ = MIC_A (in the presence of B)_/MIC_A (alone)_, and FIC_B_ = MIC_B (in the presence of A_)/MIC_B (alone)_. The antimicrobial combinatory effect was interpreted according to Odds (72), as follows: a synergistic effect if ∑FIC ≤ 0.5, no interaction if ∑FIC > 0.5 to 4, and antagonistic if ∑FIC > 4. Based on calculated FICIs, two antimicrobial agents producing the synergistic inhibition of diarrhea-causing pathogens and, simultaneously, antagonistic action on gut beneficial microbiota were identified as a combination producing a selective combinatory effect. In order to describe the synergistic and antagonistic interactions of antimicrobial agents, the minimum and maximum FICI values were used, respectively.
